# Reversal of TGF-β_1 _stimulation of α-smooth muscle actin and extracellular matrix components by cyclic AMP in Dupuytren's - derived fibroblasts

**DOI:** 10.1186/1471-2474-12-113

**Published:** 2011-05-25

**Authors:** Latha Satish, Phillip H Gallo, Mark E Baratz, Sandra Johnson, Sandeep Kathju

**Affiliations:** 1 Center for Genomic Sciences, Allegheny-Singer Research Institute, Pittsburgh, PA-15212, USA; 2 Division of Upper Extremity Surgery, Department of Orthopaedics, Allegheny General Hospital, Pittsburgh, PA-15212, USA

## Abstract

**Background:**

Myofibroblasts, a derived subset of fibroblasts especially important in scar formation and wound contraction, have been found at elevated levels in affected Dupuytren's tissues. Transformation of fibroblasts to myofibroblasts is characterized by expression of alpha- smooth muscle actin (α-SMA) and increased production of extracellular matrix (ECM) components, both events of relevance to connective tissue remodeling. We propose that increasing the activation of the cyclic AMP (cAMP)/protein kinase A signaling pathway will inhibit transforming growth factor-beta1 (TGF-β_1_)-induced ECM synthesis and myofibroblast formation and may provide a means to blunt fibrosis.

**Methods:**

Fibroblasts derived from areas of Dupuytren's contracture cord (DC), from adjacent and phenotypically normal palmar fascia (PF), and from palmar fascia from patients undergoing carpal tunnel release (CTR; CT) were treated with TGF-β_1 _(2 ng/ml) and/or forskolin (10 μM) (a known stimulator of cAMP). Total RNA and protein extracted was subjected to real time RT-PCR and Western blot analysis.

**Results:**

The basal mRNA expression levels of fibronectin- extra domain A (FN1-EDA), type I (COL1A2) and type III collagen (COL3A1), and connective tissue growth factor (CTGF) were all significantly increased in DC- and in PF-derived cells compared to CT-derived fibroblasts. The TGF-β_1 _stimulation of α-SMA, CTGF, COL1A2 and COL3A1 was greatly inhibited by concomitant treatment with forskolin, especially in DC-derived cells. In contrast, TGF-β_1 _stimulation of FN1-EDA showed similar levels of reduction with the addition of forskolin in all three cell types.

**Conclusion:**

In sum, increasing cAMP levels show potential to inhibit the formation of myofibroblasts and accumulation of ECM components. Molecular agents that increase cAMP may therefore prove useful in mitigating DC progression or recurrence.

## Background

Dupuytren's contracture (DC) is a fibroproliferative disease of the hand's palmar fascia, which can cause permanent and irreversible flexion contracture of the digits [1]. It is the most common inherited disease of connective tissues in humans [2]. Although DC is not rare, debate over its etiology has been ongoing since before its modern-day description over 120 years ago [3]. DC is known to result from changes occurring in the dermis and palmar fascia [4]. Fibroblasts are the major cell population associated with DC in all stages (both during the formation of nodules and cords) and represent an important target for therapeutic intervention. Importantly, differentiation of fibroblasts into myofibroblasts, identified by their expression of alpha-smooth muscle actin (α-SMA) [5-9], is considered to be responsible for the development of typical clinical symptoms and offers an opportunity for molecular intervention.

Myofibroblast formation is controlled by a variety of growth factors, cytokines and even mechanical stimuli [8,10]. Transforming growth factor-beta1 (TGF-β_1_) is the most important of these and has been demonstrated in Dupuytren's tissue using various techniques [11,12] along with its receptors [4]. Berndt et al. [13] showed a greater intensity of staining for TGF-β_1 _protein in proliferative nodules and colocalization of TGF-β_1 _synthesis with the myofibroblast phenotype to these regions. Furthermore, addition of TGF-β_1 _resulted in significant up-regulation of cells staining for α-SMA in primary cultures of fibroblasts derived from Dupuytren's nodule and cord tissue. It therefore seems likely that this growth factor plays a central function in the development and progression of the disease.

Surgical intervention remains the mainstay of treatment for DC, but there is a high recurrence rate after surgery [14-16]. TGF-β_1 _release might also play a significant role in the recurrence of the disease after surgical treatment. The local trauma of surgical excision and the resultant natural wound healing response will typically lead to the release of growth factors which include TGF-β_1_. Any residual tissue with a disease or pre-disease phenotype will be susceptible to stimulation, myofibroblast transformation, collagen synthesis and the formation of recurrent disease. Some studies have correlated recurrence of DC with the presence of myofibroblasts [17].

In this context, it is reasonable to hypothesize that a means of counter-acting the signaling mechanisms of TGF-β-mediated up-regulation of α-SMA and ECM gene expression in Dupuytren's tissue may provide novel approaches to the therapy of DC disease. Accordingly, we have focused our attention on cyclic AMP (cAMP), a signal transduction mediator that may interfere with TGF-β-initiated functions. The second messenger cAMP regulates fibroblast physiology in many tissues. Intracellular cAMP levels are the result of a balance between synthesis, which is regulated by G-protein-coupled receptors that stimulate (via G_s_) or inhibit (via G_i_) adenylyl cyclase (AC), and degradation, which occurs via cyclic nucleotide phosphodiesterase (PDE). Increases in cAMP influence cell growth, cell death, and differentiated cell functions, primarily (although not exclusively) by promoting phosphorylation of proteins via the activation of cAMP-dependent protein kinase A (PKA) [18]. PKA-mediated phosphorylation of cAMP-response element-binding protein (CREB) and CREB-mediated regulation of transcription via interaction with cAMP-response elements is a major pathway that alters cellular gene expression [19].

One mechanism by which cAMP may regulate fibrogenicity is via interaction with the TGF-β signaling pathway. Recent work suggests that activation of the cAMP/PKA signaling pathway inhibits TGFβ_1_-induced collagen synthesis and myofibroblast formation in cardiac and pulmonary fibroblasts [20,21]. These results suggest that overproduction of cAMP may provide a means to blunt fibrosis.

To our knowledge there have been no studies that investigate the relationship between cAMP signaling and TGF-β-mediated effects in DC disease. In this study we sought to establish the baseline functioning of cAMP and the effects of its elevation in DC-derived fibroblasts. We specifically examined alpha-smooth muscle actin, connective tissue growth factor (CTGF), as well as important components of the extracellular matrix.

## Methods

### Cell Culture

Primary cultures of fibroblasts were obtained from the surgically resected Dupuytren's contracture samples (DC), from matching specimens of normal appearing palmar fascia in DC patients (PF), and from specimens of normal palmar fascia of patients undergoing carpal tunnel surgery (CT) as previously described [22,23]. All samples were collected with the informed consent of the patient and the study protocol conformed to the ethical guidelines of the 1975 Declaration of Helsinki. All specimens were collected with the approval of the Allegheny-Singer Research Institute's institution review board involving Human Subjects (IRB protocol RC-4040) and all the patients signed the written informed consent under institutional review board approval. The cultures were maintained in MEM-α medium (Invitrogen Corporation, Carlsbad, CA) supplemented with 10% fetal bovine serum (FBS, Gemini Bioproducts, West Sacramento, CA) and 1% antibiotic-antimycotic solution (Sigma, St Louis, MO). All cultures were used at passage levels between 3-6 with no changes evident in cell morphology.

CT-, PF-, and DC- derived fibroblasts were plated onto 6-well Falcon tissue culture plates and grown until 80% confluence. Cells were quiesced for 24 hours in

MEM-α medium supplemented with 0.1% dialyzed fetal bovine serum (Gemini Bioproducts) and 1% antibiotic-antimycotic solution. After 24 hours the cells were then treated or not with TGF-β_1 _(2 ng/ml) (Peprotech, Inc. Rockyhill, NJ) and/or forskolin (Sigma) (10 μM) and incubated for 37°C for 24 hours. Cells were then washed with phosphate buffered saline (PBS) and lysed using M-PER obtained from Thermo Fisher Scientific (Rockford, IL) for protein extraction and RLT-lysis buffer (Qiagen Inc.Valencia, CA) for RNA isolation according to the manufacturer's instructions. RNA quality was assessed by A260/280 ratio using an ND-1000 spectrophotometer (Nanodrop Technologies Inc, Wilmington, DE) and by capillary electrophoresis with the Agilent 2100 bioanalyzer (Agilent technologies, Inc. Palo Alto, CA). At least three independent primary cell cultures of CT-, PF- and DC- derived fibroblasts were used in experiments involving treatment with TGF-β_1 _or forskolin. Six independent sets of CT-, PF-, and DC- derived fibroblasts were used in establishing the basal mRNA expression of specific extracellular matrix (ECM) proteins.

### Quantitative Real time RT-PCR

Total RNA isolated (RNeasy Micro Kit, Qiagen Inc., Valencia, CA) from untreated DC -, PF- and CT-derived fibroblasts was subjected to real time RT-PCR to determine the relative mRNA expression levels at baseline for fibronectin (FN1-EDA), type I collagen (COL1A2), type III collagen (COL3A1) and connective tissue growth factor (CTGF). RNA isolated from cells treated with TGF-β_1_, forskolin, and with both agents was also subjected to real time RT-PCR to determine the changes in the mRNA levels of α-SMA (ACTA2), FN1-EDA, COL1A2, COL3A1 and CTGF.

Real-time RT-PCR was performed using kits obtained from Applied Biosystems (Foster City, CA) that utilize FAM™Taqman^®^MGB probes and a Taqman^® ^Universal PCR Master Mix. Assays were performed on the above noted gene products using human GAPDH as an endogenous normalizing control. Reverse transcription was performed on 30 ng of total RNA with random primers (100 ng), gene specific primer for FN1-EDA (10 pmole) and with M-MLV-reverse transcriptase (Invitrogen Corporation, Carlsbad, CA). The primers (forward primer 5'-TAAAGGACTGGCATTCACTGATGT-3'; reverse primer - 3'-GTGCAAGGCAACCACACTGA-5') and probe (5'6 FAM-CCCTGAGGATGGAATCCATGAGCTATTCC-TAMRA 3') used for human FN1-EDA [GenBank: X07718] were designed using Primer Express software (Applied Biosystems). Primers were obtained from Integrated DNA Technologies (Coralville, IA) and Taqman probes were purchased from Applied Biosystems. In all assays the primer sets were first tested to verify that amplimers of the expected molecular weight resulted before their employment in real time RT-PCR.

Subsequent PCR amplification and detection of template was carried out using Applied Biosystems transcript-specific assays including: COL1A2 (ID- Hs01028971_m1), COL3A1 (ID-Hs00943793), ACTA2 (ID- HS00426835_g1) and CTGF (ID-Hs00170014_m1) using 15 ng of cDNA and 20x final concentration of Gene Expression Mix, which contains both forward and reverse primers adjusted to final volume of 15.0 μl. Identical reaction mixes were prepared with human FN1-EDA primers and probes. The reaction set up and the thermal cycling protocol were as previously described (23). Using the comparative critical cycle (Ct) method the expression levels of the target genes were normalized to the GAPDH endogenous control (ID-HS99999905_m1) and the relative abundance was calculated. Data were analyzed using the 7900 HT SDS software version 2.1 provided by Applied Biosystems.

### Immunoblotting

Proteins extracted were subjected to Bradford assay to determine the protein concentration. Equal quantities of proteins were separated on SDS-PAGE, transferred to a Whatman™ Protran pure nitrocellulose immobilization membrane (GE Health Care, Piscataway, NJ) and probed with antibodies specific to α-SMA (Abcam, Cambridge, MA) and fibronectin (Santacruz Biotechnology, Inc. Santa Cruz, CA) using GAPDH (Abcam, Cambridge, MA) as loading control. The membranes were conjugated with HRP-labeled secondary antibody, and the signals were detected using SuperSignal^® ^West Femto Trial Kit Prod #34094 (Thermo Scientific, Rockford, IL). The intensity of the protein bands was quantitated using NIH Image J 1.44p, available in the public domain at http://imagej.nih.gov/ij.

## Statistical Analysis

Statistical analyses were performed using two-way ANOVA utilizing GraphPad Prism 5 for Windows Version (5.04) from Graph Pad Software Inc. Utilizing the same program Bonferroni post-test to compare replicate means by row was also performed to determine the p values. P value less than 0.05 was considered significant.

## Results

### Basal mRNA expression levels of ECM proteins were significantly increased in Dupuytren-derived fibroblasts

**Figure 1:**
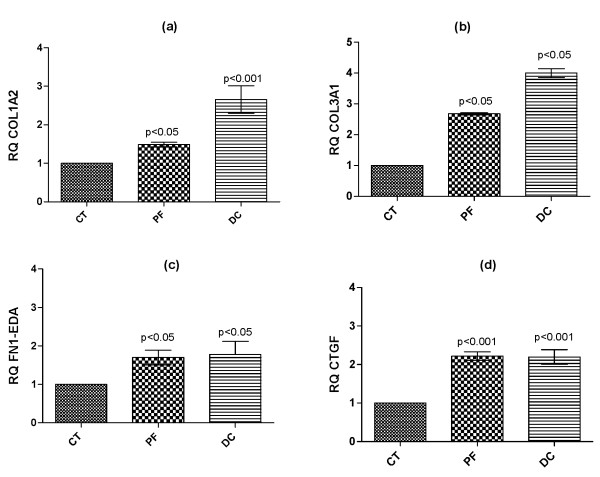
**Basal mRNA expression of ECM proteins was significantly elevated in DC-derived fibroblasts**. Real time RT-PCR was performed on RNA extracted from 6 independent primary cultures derived from CT-, PF- and DC- tissues to determine the mRNA expression levels of COL1A2 (a), COL3A1 (b), FN1-EDA (c), and CTGF (d). Values are means ± SEM of three independent experiments performed in duplicate. Statistical analyses were performed using two-way ANOVA.

We first examined the message levels of ECM proteins, namely COL1A2, COL3A1, FN1-EDA and CTGF, a matricellular protein, by qRT-PCR. Our results identified increased mRNA expression levels of all the above gene products in DC- derived fibroblasts relative to CT-derived fibroblasts (Figure 1a, b, c, d). Interestingly, PF-derived fibroblasts express these ECM components in a similar fashion to fibroblasts from active disease, suggesting that even apparently normal fascia in DC patients may harbor an incipient disease phenotype.

### Forskolin inhibited the TGF-β_1 _stimulation of α-SMA mRNA and protein

**Figure 2:**
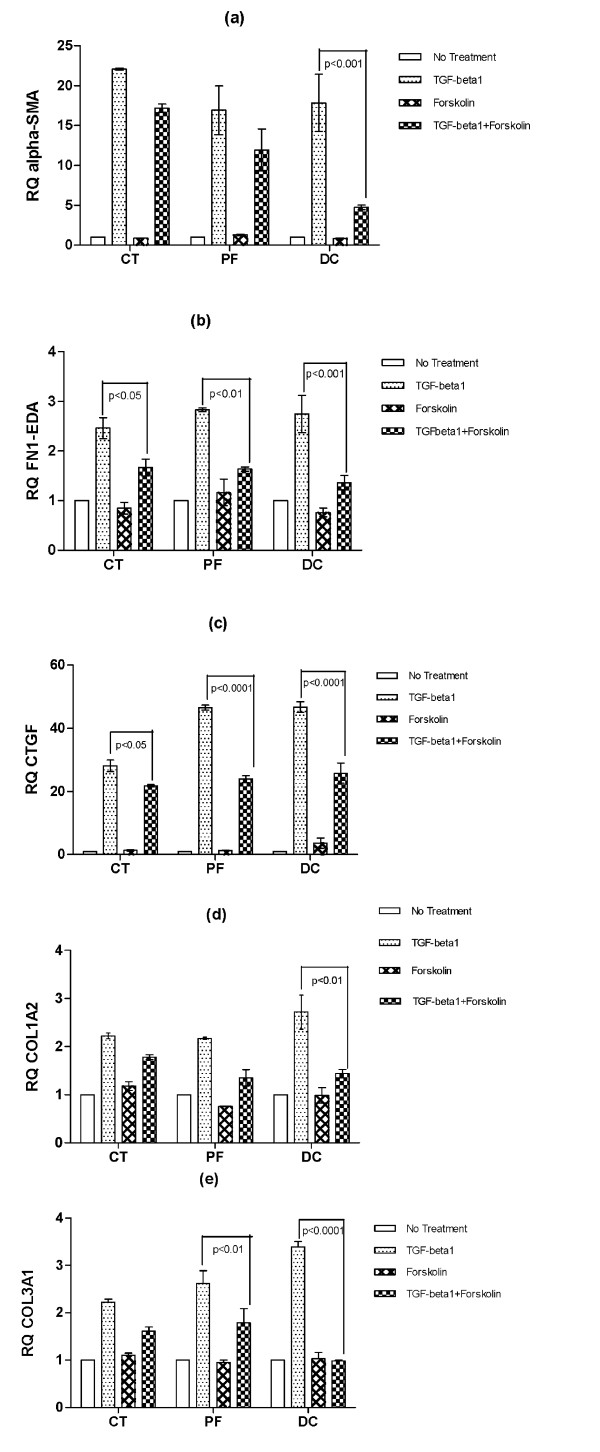
**Forskolin effectively reduced TGF-β_1 _stimulation of α-SMA, FN1-EDA, CTGF, COL1A2 and COL3A1**. Fibroblast cultures from CT-, PF- and DC- tissues were left untreated or were stimulated with forskolin (10 μM) in the presence or absence of TGF-β_1 _(2 ng/ml). Twenty-four hours later, mRNA expression levels of α-SMA (a), FN1-EDA (b), CTGF (c), COL1A2 (d) and COL3A1 (e) were analyzed by real time RT-PCR. In each experiment at least three independent cultures obtained from all the three cell types were used. Values are means ± SEM of six independent studies performed in duplicate. Statistical analyses were performed using two-way ANOVA.

**Figure 3:**
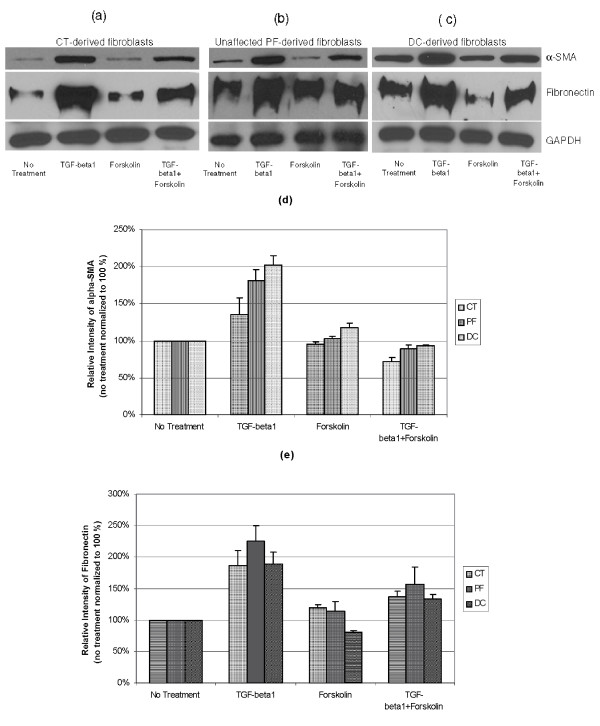
**TGF-β_1 _stimulated α-SMA and FN1-EDA protein expression were substantially reduced by forskolin in CT-, PF- and DC-derived fibroblasts**. CT-, PF- and DC-derived fibroblasts (a, b, c) grown on 6-well culture dishes were stimulated with forskolin (10 μM), TGF-β_1 _(2 ng/ml), with both agents, or were left untreated for 24 hours in MEM-α medium containing 0.1% dialyzed FBS. Whole cell lysates were collected after 24 hours and the samples were processed for Western immunoblotting with specific antibodies for α-SMA and FN1-EDA (20 μg/lane). Specificity of the modulation and identical protein loading was confirmed with a loading control GAPDH antibody. Densitometry analysis was done on protein bands obtained from three independent experiments performed in triplicate using two independent primary cultures of CT-, PF- and DC-derived fibroblasts (Figure 3d and 3e). A representative immunoblot is shown here.

Our previous findings have demonstrated an elevation at baseline of α-SMA mRNA and protein levels in DC- in comparison to CT- and PF-derived fibroblasts (Satish et al., manuscript in preparation). The present study shows that addition of TGF-β_1 _greatly augments the levels of α-SMA mRNA in CT-, PF- and DC- derived fibroblasts. To determine if increased levels of cAMP could reduce the TGF-β_1 _induced levels of α-SMA, forskolin, a well-established adenylyl cyclase (AC) activator and an inducer of cAMP in fibroblasts [20,24,25] was utilized. We found that by increasing cAMP levels there was a substantial reduction in TGF-β_1 _induced mRNA levels of α-SMA in DC- derived fibroblasts compared to TGF-β_1 _treatment alone. Although apparent reductions in TGF-β_1_-induced α-SMA mRNA levels were also observed in CT-derived fibroblasts and PF-derived fibroblasts compared with TGF-β_1 _treatment alone, the extent of these cAMP effects was significantly less than in DC-derived cells (Figure 2a). Similar significant reductions in TGF-β_1_-induced α-SMA protein levels were seen in all three-cell types by Western blot (Figure 3a-d). Forskolin by itself did not have any significant effect on α-SMA mRNA or protein levels in any cell type. These results strongly suggest that myofibroblast formation (as evidenced by α-SMA accumulation) can be significantly inhibited in DC- derived cells by increasing cAMP levels.

### Forskolin reduced the TGF-β_1 _induction of fibronectin mRNA and protein

Extracellular matrix deposition likely plays a crucial role in the fibrosis noted in DC, and previous studies have observed increased deposition of an oncofetal isoform of fibronectin (IIICS spliced variant) in DC lesional tissues and in DC-derived primary cell cultures [22]. In this study we examined FN1-extra domain A (EDA), as this isoform has shown differential expression between fibrotic versus scarless healing seen in mucosal and skin wound healing [26]. Forskolin treatment alone had no significant effect on FN1-EDA mRNA levels in any of our three cell types (Figure 2b), nor were fibronectin protein levels affected in CT- and PF-derived cells, but we did observe a significant decrease in fibronectin protein in DC- derived fibroblasts on forskolin treatment by Western blot (Figure 3a-c, e), the mechanism for which may be post-transcriptional.

We found that forskolin inhibited TGF-β_1_-induction of fibronectin mRNA to a similar degree in CT-, PF- and DC- derived fibroblasts when measured against TGF-β_1 _treatment alone (Figure 2b). This is in contrast to α-SMA, where DC-derived cells were uniquely and especially susceptible to this forskolin effect. Fibronectin protein levels in all three cell types also showed relative decrease when forskolin was added compared to TGF-β_1 _alone (Figure 3a-c, e).

### Forskolin inhibited the TGF-β_1 _induction of CTGF mRNA in PF- and DC- derived cells but not CT-derived cells

We next determined the effect of increased cAMP levels on another TGF-β_1 _target gene, CTGF. Since TGF-β may induce CTGF through several pathways, including SMAD, ras/raf/MEK/ERK, Ets-1, JNK, and protein kinase C, CTGF has long been thought to be an important mediator of its fibrotic effects [27-30]. The TGF-β_1 _induction of CTGF mRNA increase was substantially reduced by combined incubation with forskolin in PF- and DC- derived fibroblasts compared to TGF-β_1 _alone (Figure 2c). As with α-SMA, these results again suggest that the biology of fibroblasts from DC patients is exquisitely sensitive to the mitigating actions of cAMP.

### Forskolin reduced the TGF-β_1 _stimulation of Type I and Type III collagen

We next investigated the effect of increased cAMP (via forskolin treatment) on collagen expression as TGF-β is a known stimulator of collagen production [31]. We specifically examined if increased cAMP levels can abrogate TGF-β_1 _induction of type I (α-2 chain; COL1A2) and type III collagen (α-1 chain; COL3A1) expression. Forskolin alone did not have any significant effect on the relative levels of COL1A2 and COL3A1 mRNAs in any of the three cell types. Forskolin did, however, suppress the TGF-β_1 _induction of COL1A2 and COL3A1 mRNAs in CT-, PF- and DC -derived fibroblasts (Figure 2d, e). Of note, the degree of inhibition seen when TGF-β_1 _was co-incubated with forskolin was significantly greater in DC -derived cells than in the CT- or PF-cells. Since increased collagen deposition is a hallmark of DC disease, these results again suggest that mechanisms to elevate cAMP may be useful adjunctive therapies to counteract the fibrotic phenotypes of DC cells.

## Discussion

Dupuytren's contracture, fibrosis in the palmar fascia of the hand, is a fibroproliferative disorder that can impose severe functional damage eventually leading to disability of the hand in affected individuals [32]. Efforts have been made to control the fibrosis seen in DC using various non-surgical treatment strategies but with limited success [33]. Injectable collagenase clostridium histolyticum [34] to treat DC shows potential promise but its clinical application has thus far elicited a varied response among hand surgeons. Alternative treatment options including non-surgical molecular therapeutic agents to prevent progression and recurrence of DC disease are still wanting.

Because myofibroblast formation and activity have been linked to the etiology of both primary and recurrent DC, molecular interventions that interfere with myofibroblastic functions may offer a novel avenue of therapy. A number of such interventions have been proposed and essayed. Glucocorticoids have been shown to increase apoptosis of Dupuytren's-associated fibroblasts, and to reduce the abundance of TGF-β_1 _and fibronectin CS1 in myofibroblast-populated stroma in DC nodules injected with depomedrone [35,36]. Repeated intralesional injection of DC nodules (not cords) with triamcinolone did show some regression of the nodules [37] but some 50% of patients developed recurrence or progression of the disease within the window of the study. Whether such an approach would succeed in more advanced disease with actual cord formation is unclear.

Another agent that acts against myofibroblasts that has been used in DC is 5-fluorouracil (5-FU). Treatment of DC-derived fibroblasts with 5-FU inhibited their proliferation and their differentiation to myofibroblasts [38]. However, clinical use of 5-FU at the time of surgery resulted in no difference between treated and untreated digits as determined by joint angle measurements [39], leaving its clinical utility open to question.

It has been observed in rat cardiac fibroblasts and in a human pulmonary fibroblast-derived cell line that elevation of cAMP can inhibit cellular proliferation and differentiated functions (such as collagen synthesis). These observations suggested that a similar approach might favorably alter fibroblast/myofibroblast behavior in the setting of Dupuytren's contracture. We therefore sought to determine if increased cAMP levels could inhibit TGF-β_1_-induced myofibroblast formation (as indicated by α-SMA accumulation) and ECM production in DC-derived cells. TGF-β_1 _was chosen as a test stimulatory cytokine as it has been implicated in the pathogenesis of DC [2,4,40].

Multiple interesting observations have arisen from these experiments. When assaying for basal levels of expression of α-SMA and ECM proteins in our three cell types, it is clear that PF-derived cells more closely resemble DC-derived cells than control CT-derived cells in all four gene products tested. This suggests that, although obtained from phenotypically normal fascia, PF-derived cells may already exhibit a disease phenotype at the cellular level. Such an observation is consistent with our total expressomic analyses of DC- and PF- versus CT-derived fibroblasts, wherein we find that global gene expression patterns of PF-cells closely resemble (but are not identical to) DC-derived cells and vary sharply from CT-derived cells (Satish et al., manuscript in preparation).

We also found that TGF-β_1_, as expected, increased expression levels of all gene products assayed significantly, whereas cAMP elevation (as induced by forskolin treatment) alone had minimal effect. cAMP was, however, in all instances able to dramatically blunt the effects of TGF-β_1_. DC-derived cells were particularly susceptible to cAMP action, generally exhibiting more inhibition of gene expression by cAMP action than PF- or CT-cells. These observations suggest that agents to elevate cAMP may well be able to suppress the differentiation of DC-fibroblasts to a myofibroblast phenotype, and to mitigate the abnormal ECM deposition that would then typically ensue. Although forskolin (or other similar agents) may be impractical to deliver directly to DC-affected tissues over the long periods of time in which the disease develops or progresses, we postulate that molecular therapeutic approaches administering activated adenylyl cyclase, possibly by a gene therapy approach, may accomplish the same effects. Successful use of adenylyl cyclase to inhibit myofibroblast formation and function has been demonstrated in cardiac and pulmonary cells [20,21].

A particular point of interest in this study is the examination of the behavior of CTGF in our three cell types. CTGF has been described as a co-factor to TGF-β by enhancing ligand-receptor binding in activated cells [41]. Studies in various cell populations have also demonstrated roles for CTGF in the TGF-β-dependent induction of fibronectin, collagen and tissue inhibitor of metalloproteinase-1 (TIMP-1) [42-44]. A recent study by Sisco et al. [45] showed that antisense inhibition of CTGF could limit hypertrophic scarring *in vivo *without affecting the outcome of wound closure. To our knowledge this report for the first time demonstrates increased basal expression levels of CTGF in PF- and in DC-derived fibroblasts compared to CT-derived cells, and this relative increase is enhanced by addition of TGF-β_1_. Further, we also find that elevated cAMP levels most successfully reduce this increased CTGF mRNA expression in DC-derived fibroblasts. This report thus points to a potential role for CTGF in the etiopathology of DC, and suggests that measures to target its expression or function (including agents that elevate cAMP) may usefully limit fibrosis in Dupuytren's contracture.

The observations reported herein do not directly identify the precise mechanisms by which increased cAMP levels inhibit myofibroblast formation. Recent data indicate that cAMP acts in a PKA-dependent manner to inhibit TGF-β/Smad signaling and gene activation by disruption of transcriptional cofactor binding in human keratinocytes [46]; it is possible that similar mechanisms are at work in DC-fibroblasts, and are being investigated. Moreover, we are in the process of delineating the migratory and contractile behavior of DC-derived fibroblasts when cAMP levels are increased. Demonstration of a change in these mechanocellular properties would provide even more evidence of the utility of a cAMP-based approach as an anti-fibrotic measure in Dupuytren's contracture.

## Conclusion

In summary, increasing cAMP levels show potential to inhibit the formation of myofibroblasts and accumulation of ECM components. Molecular agents that increase cAMP may therefore prove useful in mitigating DC progression or recurrence.

## Competing interests

The authors declare that they have no competing interests.

## Authors' contributions

LS conceived the study. LS and SK discussed and designed the study. LS, PG, SJ performed the experiments. LS and SK drafted the manuscript. LS, SK and MEB critically reviewed manuscript. All authors read and approved the final manuscript.

## Pre-publication history

The pre-publication history for this paper can be accessed here:

http://www.biomedcentral.com/1471-2474/12/113/prepub
